# Chemical emasculation in cowpea (*Vigna unguiculata* (L.) Walp.) and dicotyledonous model species using trifluoromethanesulfonamide (TFMSA)

**DOI:** 10.1007/s00497-023-00469-4

**Published:** 2023-05-25

**Authors:** Yuka Sekiguchi, Benjamin Ewa Ubi, Takayoshi Ishii

**Affiliations:** 1 Graduate School of Sustainability Science, Tottori University, 4-101 Koyama Minami, Tottori, 680-8550 Japan; 2 Arid Land Research Center, Tottori University, 1390 Hamasaka, Tottori, 680-0001 Japan; 3 Department of Biotechnology, Ebonyi State University, PMB 053, Abakaliki, Nigeria

**Keywords:** Pollination control, Pollen sterility, Neglected crops, Male gametocide, Dryland crop, Hybrid seed production

## Abstract

Hybridization plays an indispensable role in creating the diversity associated with plant evolution and genetic improvement of crops. Production of hybrids requires control of pollination and avoidance of self-pollination for species that are predominantly autogamous. Hand emasculation, male sterility genes or male gametocides have been used in several plant species to induce pollen sterility. However, in cowpea (*Vigna unguiculata* (L.) Walp), a self-pollinated cleistogamous dryland crop, only hand emasculation is used, but it is tedious and time-consuming. In this study, male sterility was effectively induced in cowpea and two dicotyledonous model species (*Arabidopsis thaliana* (L.) Heynh. and *Nicotiana benthamiana* Domin) using trifluoromethanesulfonamide (TFMSA). Pollen viability assays using Alexander staining showed that 30 ml of 1000 mg/l TFMSA with two-time treatments of one-week interval at the early stage of the reproductive phase under field or greenhouse conditions induced 99% pollen sterility in cowpea. TFMSA treatment induced non-functional pollen in diploid *A. thaliana* at two-time treatment of 10 ml of 125–250 mg/l per plant and *N. benthamiana* at two-time treatment of 10 ml of 250–1000 mg/l per plant. TFMSA-treated cowpea plants produced hybrid seeds when used as the female parent in crosses with non-treated plants used as male parents, suggesting that TFMSA had no effect on female functionality in cowpea. The ease of TFMSA treatment and its effectiveness to induce pollen sterility in a wide range of cowpea genotypes, and in the two model plant species tested in this study, may expand the scope of techniques for rapid pollination control in self-pollinated species, with potential applications in plant breeding and plant reproduction science.

**Supplementary Information:**

The online version contains supplementary material available at 10.1007/s00497-023-00469-4.

## Introduction

In self-pollinated plant species, seeds are produced by self-fertilization, whereas in cross-pollinated (allogamous) species outcrossing results in hybrid progeny (Ter Steeg et al. 2022). However, the division between self-pollinators and cross-pollinators is not entirely rigid. Some species undergo obligate self-fertilization, other self-pollinators can occasionally exhibit some degree of outcrossing; viable seeds may also result from selfing in some allogamous species (Ter Steeg et al. 2022). Natural mechanisms such as dioecy, dichogamy (protandry and protogyny), self-incompatibility and male sterility may hinder self-pollination in some plant species. In monoecious species such as maize, where the pistillate and staminate flowers are separated on the same plant, pollination control is relatively easy, enabling strategies to facilitate hybridization between parental lines to exploit heterosis (Crow 1998). In self-pollinated species such as cleistogamous cowpea, pollination takes place before the flower is open, thereby ensuring self-pollination. Pollination control in such species can be a major challenge; genetic systems and artificial methods to induce male sterility are needed to reduce the laborious and time-consuming hand emasculation for hybrid seed production in these species.

Several methods to induce male sterility to enable hybrid seed production in self-pollinated plant species have been reported (Chang et al. 2016; Perez-Prat and van Lookeren Campagne 2002). Male sterility (the absence of functional pollen for self-fertilization) has been widely used for hybrid seed production (Du et al. 2020; Lasa and Bosemark 1993). Genetic male sterility is governed by nuclear genes, whereas cytoplasmic male sterility (CMS) is due to a mutation in the mitochondrial genome (Kaul 1988). CMS has been widely exploited for hybrid seed production, with success driven by such factors as availability of CMS mutants within the crop species, presence of restorer (nuclear) genes to restore fertility of the CMS lines and the absence of a deleterious effect of the CMS mutation on yield (Chang et al. 2016; Eckardt 2006; Perez-Prat and van Lookeren Campagne 2002). In cowpea, reports of male sterility lines are relatively few: Adu-Dapaah et al. (1999) and Odeigah et al. (1996) obtained completely sterile cowpea mutants from spontaneous and induced mutations, which failed to set seed when crossed with fertile parents.

Hot water and chemical emasculation methods have been reported for bulk emasculation in some crop species (Abd El-Aty et al. 2022; Jodon 1938; Lv et al. 2018; Stetter et al. 2016). A variety of chemicals, called male gametocides or chemical hybridizing agents (CHA), have been used for effective induction of male sterility to widen the scope of hybrid seed production in crop species such as rice, maize, wheat, cotton and sunflower (Ali et al. 1999; Colombo and Galmarini 2017; Ghebrehiwot et al. 2015; Mogensen and Ladyman 1989; Naylor and Davis 1951; Razzaq et al. 2016; Tripathi and Singh 2008). However, some of these promising chemicals may be protected by patents, and/or could be toxic to human health or the environment. Additional methods of generating male-specific sterility without adverse effects on female functionality could expand the scope of techniques for rapid and effective pollination control, especially in self-pollinated crops such as cowpea.

Trifluoromethanesulfonamide (TFMSA) is a common chemical used as catalyst, solvent for electron paramagnetic resonance, acid titrant and peptide synthesis. Hazard statements in Globally Harmonized System of Classification and Labelling of Chemicals (United Nations GHS Rev. 9, 2021) are classified as combined code with H315 + H320 indicates skin and eye irritation (https://pubchem.ncbi.nlm.nih.gov/compound/79001). Trifluoromethanesulfonamide (TFMSA) is a common chemical and not yet found to be a plant hormone (Loussaert 2004; Moskalik and Astakhova 2022; Shainyan and Tolstikova 2013). TFMSA has been studied as a male sterility-specific CHA with potential applications in maize (Loussaert 2004) and sorghum (Hodnett and Rooney 2018). TFMSA induces up to 100% male sterility in tested species under greenhouse and field conditions, depending on the dosage and timing of application. TFMSA has no adverse effects on other plant functions including germination of the resulting hybrid seeds in sorghum and maize (Hodnett and Rooney 2018; Loussaert 2004). Metabolite profiling in maize indicated that TFMSA inhibits proline accumulation in the developing anthers (Loussaert 2004). However, to the best of our knowledge, reducing pollen fertility using TFMSA has not been reported in dicotyledonous species.

Cowpea (*Vigna unguiculata* (L.) Walp 2*n* = 2*x* = 22) is a legume of great importance for food and nutrition security. The crop originated from the dry regions of West Africa, and is tolerant of drought and high temperatures. Basic research on this crop is rapidly increasing for the improvement of cowpea for future food security in drylands (Che et al. 2021; Edet and Ishii 2022; Ishii et al. 2020; Lonardi et al. 2019; Spriggs et al. 2018). However, to enhance hybrid seed production in cowpea, a cleistogamous species, an effective male-specific sterility induction technique is needed. The use of TFMSA to induce male sterility in cowpea and other dicotyledonous species therefore warrants examination.

Model plant species have been used extensively in research including genomic studies, because of their small plant size, relatively small genome size and short growth cycle. Two model dicot species, *Arabidopsis thaliana* (L.) Heynh. and *Nicotiana benthamiana* Domin, can be used to efficiently test the effect of a CHA on plant reproductive functions and phytotoxicity.

This study had the following objectives: (i) to investigate the use of TFMSA for male sterility induction in cowpea, *A. thaliana* and* N. benthamiana*; (ii) to determine the optimum dosage and timing of application for sterility induction; and (iii) to confirm female functionality in induced male-sterile cowpea.

## Material and methods

### Plant materials and growth conditions

A total of 62 cowpea accessions, plus diploid *A. thaliana* (140,931; Col-0, 2*n* = 2*x* = 10), tetraploid *A. thaliana* [N3151; Col-0, 2*n* = 4*x* = 20 (Comai et al. 2000)] and *N. benthamiana* (2*n* = 4*x* = 38) were used in this study (Table S1). Accession ID of the NARO Genebank Japan (https://www.gene.affrc.go.jp/databases_en.php) was indicated. Seeds of 60 cowpea accessions were sown at the same time in an experimental field of the Arid Land Research Center, Tottori, Japan, during the 2021 growing season (May to October). Cowpea accessions IT86D-1010 and IT97K-499-35 were grown in a greenhouse (16 h day at 26 °C/8 h night at 18 °C) in 3.5-l pots (diameter 18 cm, height 20.5 cm) filled with granular culture soil (Nippi-Engei-Baido, Nihon Hiryo Co. Ltd., Tokyo, Japan), one plant per pot, 3 replications, 36 plants in total in 2020 and 2021. *Nicotiana benthamiana* plants were germinated and grown in a growth chamber under the same conditions as for cowpea in the greenhouse, in 7.5 cm × 6.5 cm pots filled with a planting soil mixture (Takii-Tanemaki-Baido, Cainz Co., Saitama, Japan), one plant per pot, 3 replications, 18 plants in total, in 2021. *Arabidopsis thaliana* plants were grown in pots (7.5 cm × 6.5 cm) under the same conditions as *N. benthamiana* for 5–7 days after germination, then in a vernalization room (9 h day/15 h night, both at 4 °C) for one month, and then in a plant growth room (9 h day at 26 °C/15 h night at 18 °C) until flower bud formation, and finally in a flowering room (12 h day at 26 °C/12 h night, at 18 °C) for flowering.

### TFMSA treatment

Trifluoromethanesulfonamide (Tokyo Chemical Industry Co. Ltd., Tokyo, Japan) was dissolved in an aqueous solution of Approach BI (0.1% v/v; Maruwa Biochemical Co. Ltd., Tokyo, Japan) as a spreading agent, to produce concentrations of 0, 62.5, 125, 250, 500 and 1000 mg/l (w/v). Cowpea plants were treated with TFMSA after all of them were confirmed to be flowering and the flowers (including buds) and pods were removed; diploid *A. thaliana,* tetraploid *A. thaliana* and *N. benthamiana* were treated when the first flower buds were observed.

### Evaluations on optimal timing

To test the optimal timing for TFMSA application to cowpea, IT97K-499-35 grown in the field was treated with 30 ml of 1000 mg/l TFMSA solution per plant, one to five times, starting the first treatment when the first fully expanded leaf was observed, which usually happened approximately 5 weeks before anthesis (Fig. S1a). The plant height, number of branches, peduncles, pods and seeds were determined for each plant at senescence.

### Evaluations on optimal dosage

To test the optimal dosage, concentrations of 30 ml of 0, 62.5, 125, 250, 500 and 1000 mg/l (w/v) TFMSA solution per plant were sprayed onto each cowpea plants per treatment in the greenhouse, and 10 ml of 0, 62.5, 125, 250, 500 and 1000 mg/l (w/v) TFMSA solution per plant was sprayed onto each *A. thaliana* and *N. benthamiana* plant, using a hand sprayer. One flower from each plant was collected and two anthers from each flower were dissected for fertility analysis. Flowers were collected and anthers were fixed and assessed as described below. Exact amount of the TFMSA solution sprayed to the plant (above ground) was tested in cowpea and *N. benthamiana.* Thirty milliliters ml or 10 ml of solution was sprayed on to the cowpea or *N. benthamiana,* respectively*.* The increased weight after the application of the TFMSA solution was measured. Pollen grains of the cowpea accessions were dissected from anthers collected 7 days after the second TFMSA application, for detailed observation of pollen viability resulting from the different dosages applied, and assessed as described below.

### Evaluations on wider cowpea accessions in field

The effect of TFMSA treatment under field condition was investigated using IT86D-1010 and 41 other cowpea accessions, four plants per accessions, in 2021. A single treatment with TFMSA solution at 30 ml of 1000 mg/l (w/v) per plant was applied in the field after all of them were confirmed to be flowering and the flowers (including buds) and pods were removed. One week after treatment, the flowers were collected when the formation of the new flower occurs, and anthers were fixed for pollen viability analysis. Two flowers were selected randomly from 4 plants in one accession for pollen viability analysis (Fig. S2). Three of the accessions received further field testing involving applying 30 ml of 1000 mg/l (w/v) TFMSA solution per plant two times, a week apart, when anthesis starts, in 2021.

### Pollen viability analysis

Anthers from each plant of all species investigated in this study were collected from the flower buds about 24 h before flower opens by judging the color and the size of the flower buds, fixed with 6:3:1 ethanol/chloroform/glacial acetic acid (v/v/v) for 3 days at room temperature, and then stored at 4 °C until use. Pollen viability was evaluated with the Alexander staining method (Alexander 1969; Peterson et al. 2010). The Alexander staining solution was prepared as follows: 10 ml of 95% ethanol, 1 ml of malachite green (1% in 95% ethanol solution), 50 ml of distilled water, 25 ml of glycerol, 5 ml of fuchsin acid (1% aqueous solution), 0.5 ml of orange G (1% aqueous solution), 4 ml of glacial acetic acid. The solution was mixed and stored at room temperature in the dark until used. Fixed anthers were placed in a 1.5 ml Eppendorf tube containing 200 µl Alexander staining solution, and placed on a heating block at 95 °C for 5 min. The anthers were then placed on a glass slide, and the staining solution (10–30 µl) was added dropwise. The pollen grains were either spread by dissecting the anthers with needles or the intact anther was squashed between the microscope slide and the cover glass (22 × 22 mm).

Specimens were photographed under a VHX-7000 digital microscope (Keyence Corporation, Osaka, Japan) for the pollen viability analysis. Pollen grains were stained as red when viable, stained blue when sterile　by Alexander staining. Viable and sterile pollen grains were counted with the machine learning software “ilastik” (Berg et al. 2019), using the workflow “Pixel Classification + Object Classification.” Assessment of the pollen grains began by inputting teacher data, i.e., by identifying the color and size of the viable and sterile pollen grains and their backgrounds in the training set of pollen grain photos. Next, all of the pollen grain photographs were evaluated regarding the presence of viable and sterile pollen grains, as judged by the software. The result of the number of pollen grains was compared with the result from counting manually by human to confirm the effectiveness of the software. There was no significant difference between the manual counting and software counting. When pollen grains were clustered, photo-editing software, Adobe Photoshop (Adobe Inc. CA. USA), was used to visually check their status, instead of using the “ilastik” method.

### Recovery of pollen fertility in cowpea

Cowpea accession IT86D-1010 was grown in a greenhouse (16 h day at 26 °C/8 h night at 18 °C) in 3.5-l pots (diameter 18 cm, height 20.5 cm) filled with granular culture soil, one plant per pot, 3 replications, 36 plants in total in 2020. The same series of TFMSA dosages as described above was applied two times, a week apart, when anthesis starts. The treated plants were grown until senescence, and the number of pods was determined at 7, 14, 21 and 61 days after the second treatment.

### Confirmation of cowpea female functionality

Plants of IT97K-499–35 (white seed coat) were treated with 30 ml of 1000 mg/l (w/v) TFMSA solution two times, a week apart, when anthesis starts and were hand pollinated with pollen from 29 accessions (full brown seed) (Table S2). Two hybrid F_1_ seeds from each cross were sown in 2021 in the field, and the seed coat color of the F_2_ seeds was checked to confirm true hybridity. The black seed coat was expected in ratio of 100% as the result of cross from parent with white seed coat and parent with full brown seed coat (Herniter et al. 2019).

### Data analysis

Data were analyzed with IBM SPSS software (IBM). Chi-squared test and student t test were used to assess the significance of difference in percentages of fertile pollen grains in IT86D-1010 plants and the effects of TFMSA treatments on *N. benthamiana*, diploid *A. thaliana* and tetraploid *A. thaliana* and plant characteristics of field grown IT97K-499-35 with at least three-time replication for each analysis.

## Results

### Induction of male sterility in cowpea

**Fig. 1:**
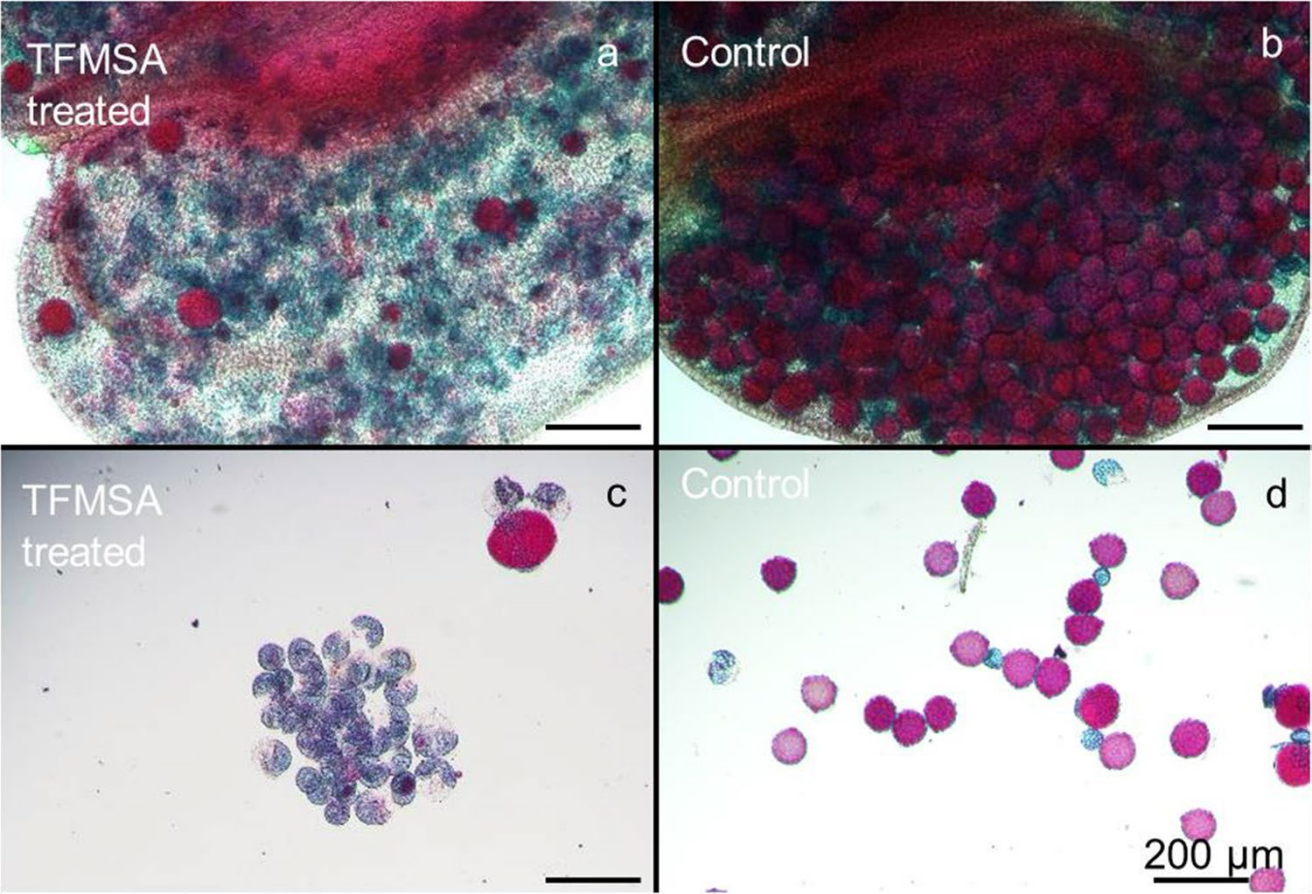
Alexander staining of anthers of cowpea IT86D-1010, treated twice a week apart with 0 or two-time treatment with 30 ml of 1000 mg/l TFMSA per plant, and sampled 7 days after the second treatment. **a** Anther of cowpea treated with two-time treatment with 30 ml of 1000 mg/l (60 ml in total) per plant. **b** Anther of cowpea treated with two-time treatment with 30 ml of 0 mg/l per plant (control). **c** Suspended pollen grains from anther in (**a**). **d** Suspended pollen grains from anther in (**b**)

**Fig. 2:**
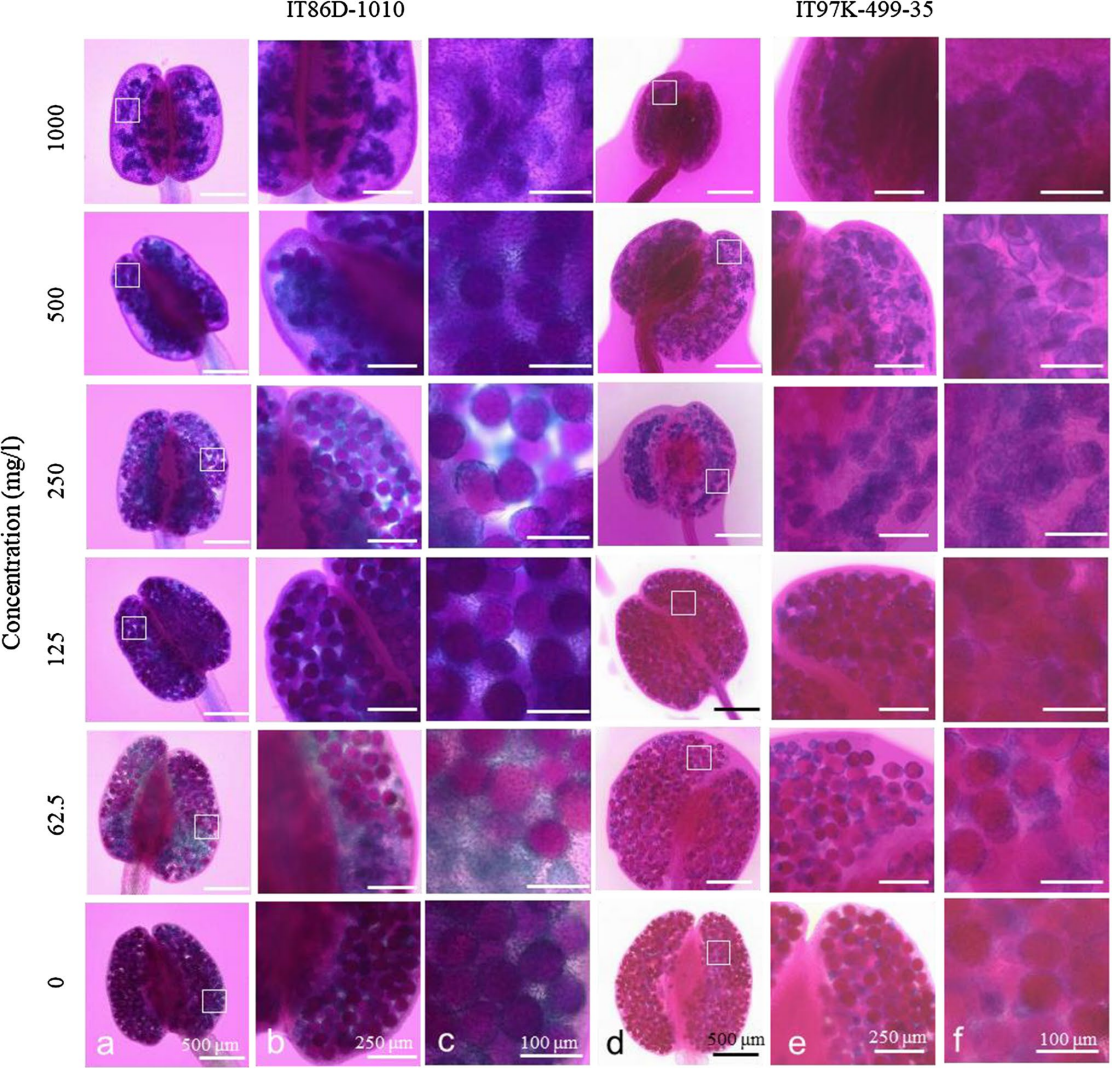
Alexander staining of anthers from cowpeas treated two times with different dosages of TFMSA, sampled 7 days after second treatment. **a** Accession IT86D-1010. **b** Double magnification of areas indicated by white squares in (**a**). **c** Five-time magnification of areas indicated by white squares in (**a**). **d** Accession IT97K-499–35. **e** Double magnification of areas indicated by white squares in (**d**). **f** Five-time magnification of areas indicated by white squares in (**d**)

In the field testing of timing and number of treatments, TFMSA delayed growth of cowpea accession IT97K-499-35, with the degree of effect directly dependent upon the number of treatment applications (Fig. S1b). The plant height decreased with the five-time treatment indicating the negative effect of TFMSA on plant growth. The mean number of pods and seeds per plant obtained from all the treatments was significantly (*p* < 0.05 to < 0.01) lower than in control plants (Table 1). In plants which received two to five treatments of 30 ml of 1000 mg/l (w/v) TFMSA solution per plant, few or no seeds were formed, whereas those treated once had relatively many pods and seeds (Table 1). The plants that received four-time treatments of 30 ml of 1000 mg/l (w/v) TFMSA solution per plant formed average of 1.5 pods. It is possible that those pods formed on plants were due to pollen blown by wind or carried by insects from the control plants grown nearby or unsuccessful induction of sterility by yet unknown factors under fileld condition. These results suggest that two applications, one week apart, before anthesis, are the most suitable treatment for induction of nearly 100% pollen sterility in cowpea, whereas the one-time treatment one week before anthesis apparently did not affect the flowers sufficiently to achieve the desired degree of male sterility. Therefore, two applications of 30 ml of 1000 mg/l (w/v) per plant, a week apart before anthesis, are the optimal TFMSA treatment for emasculation of cowpea. The exact amount of the TFMSA solution sprayed onto plants were tested in cowpea IT86D-1010 and IT97K-499-35*.* Exactly, 10.4 and 11.0 ml of solution remained on the cowpea plants after applying the 30 ml solution in cowpea IT86D-1010 and IT97K-499-35, respectively (Table S3), while 30% of solution remained on the plant surface. Thus, 10.4 and 11.0 mg of TFMSA affected cowpea in case of 1000 mg/l concentration of TFMSA solution. Alexander staining showed that 30 ml of 1000 mg/l (w/v) TFMSA treatment resulted in the preponderance of blue-stained pollen grains from anthers of IT86D-1010 plants, indicating a high degree of pollen sterility, whereas pollen grains from control plants stained red, indicating viability (Fig. 1). Application of 30 ml of 500 or 1000 mg/l TFMSA solution per plant induced a high degree of pollen sterility in both cowpea genotypes (IT86D-1010 and IT97K-499-35), as indicated by blue-stained grains (Fig. 2). A high degree of pollen sterility was also observed in the 250 mg/l TFMSA solution treatment in IT97K-499-35, and many non-functional (blue-stained) pollen grains were also observed in both cowpea genotypes at the 30 ml of 62.5 and 125 mg/l TFMSA solution treatments (Fig. 2). This suggested that application of TFMSA induced pollen sterility in cowpea the most when applied at the rate of 30 ml of 1000 mg/l (w/v) TFMSA solution per plant in both genotypes.

**Table 1:** Plant characteristics of field grown IT97K-499-35 after 1–5 treatments with 30 ml of 1000 mg/l per plant

Number of treatments	Mean number				
	Plant height (mm)	Branches	Peduncles	Pods	Seeds
5	226	6.0	33	0**	0**
4	263	6.0	41	1.5*	9.5**
3	318	6.3	48	0**	0**
2	280	7.0	37	2.0*	1.0**
1	313	7.0	71	4.3*	22**
Control	401	6.0	42	16	121

*, **Significantly different from the control at the 5% and 1% levels, respectively, Student *t* test. *n* = 2 or 3, for each treatment level

The percentages of fertile pollen grains in IT86D-1010 plants treated with TFMSA differed significantly (*p* < 0.01) from the control treatment (Table 2). There were variations of percentages of fertile pollen between the plants within treatments at 30 ml of 125, 250 and 500 mg/l. The treatment of 30 ml of 1000 mg/l (w/v) TFMSA solution effectively induced 99% of pollen sterility in individual plants. Therefore, it is possible to sterilize anthers more effectively by treating at 30 ml of 1000 mg/l (w/v) TFMSA solution.

**Table 2:** Number of viable and sterile pollen grains from cowpea IT86D-1010 plants grown in a greenhouse, treated two times with different dosages of TFMSA

TFMSA (concentration mg/l)	Number of plants	Total pollen grains	Number of viable pollen grains	Number of sterile pollen grains	Overall % sterile	Significant difference from control
Control	1	298	239	59	19	
	2	221	173	48		
62.5	1	200	95	105	63	**
	2	184	49	135		
125	1	279	115	164	59	**
	2	230	0	230		
	3	229	188	41		
250	1	194	0	194	76	**
	2	265	147	118		
	3	163	1	162		
500	1	191	0	191	68	**
	2	387	63	324		
	3	268	211	57		
1000	1	205	1	204	99	**
	2	192	1	191		
	3	214	4	210		

One flower from each plant was collected and two anthers from each flower were dissected. Viable and sterile pollens were counted as total pollen number of two anthers

**Significantly different from the control at 1%, Chi-squared test

### Evaluation of wider cowpea accessions in field

In the field trial, complete pollen sterility was observed in 38 accessions, one had partially functional pollen, and the pollen viability of three accessions was completely unaffected after a one-time treatment with 30 ml of 1000 mg/l (w/v) TFMSA solution (Fig. S2). These three accessions were retested with the two-time treatment, and male sterility was induced (data not shown).

### Recovery of pollen fertility

All TFMSA-treated cowpea plants in the pollen-recovery test formed new pods by 7 days after the second treatment (Table S4), presumably attributable to non-affected flowers emerging after the first treatment. This is consistent with the earlier results under field conditions, in which TFMSA treatment one week prior to cowpea flowering failed to prevent pod setting (Table 1). The number of pods did not change much in TFMSA-treated plants during 14–21 days. Pod number tends to increase with TFMSA-treated plants with 30 ml application at concentration of 0, 62.5, 125, 250 mg/l at 61 days after treatment. In contrast, pod number did not increase in plants treated with 30 ml of 1000 mg/l at 61 days after treatment. Pod number at 30 ml of 500 mg/l treatment per plant was at an intermediate level. Pod formation indicates that pollen fertility was restored at 30 ml of 62.5–500 mg/l TFMSA treatment, while 30 ml of 1000 mg/l TFMSA solution treatment maintained pollen sterility for at least two months (Table S4). Newly formed pods were indicative of the recovery of pollen after 61 days after the second treatment.

### Plant growth and TFMSA male sterility in *A. thaliana* and *N. benthamiana*

**Fig. 3:**
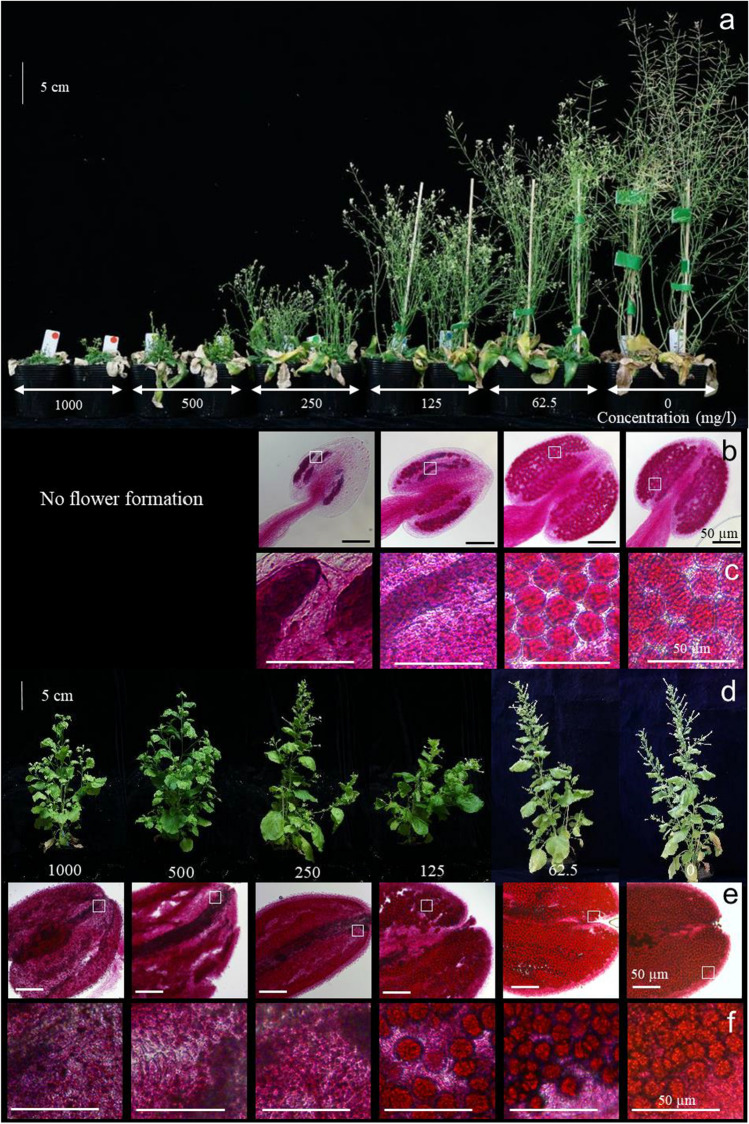
Effects of TFMSA treatment on plant growth and pollen. **a** *A. thaliana* plants 21 days after second treatment with different dosages. **b** Alexander staining of anthers of plants from (**a**), 7 days after second TFMSA treatment. **c** Magnification of areas indicated by white squares in (**b**). **d** *N. benthamiana* 30 days after second treatment with different dosages. **e** Alexander staining of anthers of plants from (**d**), 7 days after second TFMSA treatment. **f** Magnification of areas indicated by white squares in (**e**)

Plant growth of diploid *A. thaliana* and *N. benthamiana* was significantly affected by TFMSA treatment (Fig. 3a, d). Diploid *A. thaliana* treated with 10 ml of 500 and 1000 mg/l TFMSA solution per plant did not form flowers. No functional pollen was formed in the flowers of diploid *A. thaliana* treated with 10 ml of 125 or 250 mg/l TFMSA solution per plant, nor in flowers of *N. benthamiana* treated with 10 ml of 250–1000 mg/l (Fig. 3b, c, e and f). Plant height decreased in a dosage-dependent manner in diploid *A. thaliana* and in *N. benthamiana*, suggesting inhibitory effects of TFMSA on the growth of both species (Table 3). This effect was more pronounced in diploid *A. thaliana* than in *N. benthamiana*. The number of branches did not change in relation to dosage in either species. The number of siliques or fruits without seeds decreased significantly in TFMSA-treated *N. benthamiana* compared with the control, but first increased and then decreased in diploid *A. thaliana* treated with increase in dosage of TFMSA (Table 3). In diploid *A. thaliana*, the number of siliques with seeds was 44.3 in control plants, but only 5.5 in plants treated with 10 ml of 62.5 mg/l TFMSA per plant, and 0 at higher dosages. The number of fruit in *N. benthamiana* tended to decrease as the TFMSA dosage increased, which is consistent with the observation that non-fertilized flowers were dropping off the plants. The exact amount of the TFMSA solution sprayed onto plants were tested in *N. benthamiana*. Exactly, 2.7 ml of solution remained on the *N. benthamiana* plants after applying the 10 ml solution (Table S3), while 30% of solution remained on plat surface. Thus, 2.7 mg of TFMSA affected *N. benthamiana* in case of 1000 mg/l concentration of TFMSA solution. These findings suggest that TFMSA applied to *N. benthamiana* and diploid *A. thaliana* induced pollen sterility and suppressed plant growth in a dosage-dependent manner. This suggests that an optimized dosage may induce pollen sterility without serious growth retardation in these dicotyledonous model species.

**Table 3:** Effects of TFMSA treatments on *N. benthamiana,* diploid *A. thaliana* and tetraploid *A. thaliana*

TFMSA (concentration mg/l)	Species	Mean plant height (cm)	Mean number of		
			Branches	Silique or fruit with seeds	Silique or fruit without seeds
0	*N. benthamiana*	64.3	23.7	55.3	26.3
	dip.^a^ *A. thaliana*	46.0	5.75	44.3	3.50
	tet.^b^ *A. thaliana*	45.3	8.33	16.3^†^	11.3
62.5	*N. benthamiana*	64.2	23.7	24.7*	5.67**
	dip. *A. thaliana*	35.3*	9.75*	5.50**	26.3**
	tet. *A. thaliana*	43.5	8.33	20.7	16.7
125	*N. benthamiana*	62.1	20.3	16.7**	3.33**
	dip. *A. thaliana*	22.8**	12.8**	0**	14.8**
	tet. *A. thaliana*	39.3^††^	8.67	11.3	25.7
250	*N. benthamiana*	61.7	22.7	10.0**	4.33**
	dip. *A. thaliana*	12.5**	14.0**	0**	6.00
	tet. *A. thaliana*	41.2^††^	9.00	2	28.7*^††^
500	*N. benthamiana*	56.0	19.0	3.33**	0.333**
	dip. *A. thaliana*	5.88**	10.5**	NA	NA
	tet. *A. thaliana*	36.7^††^	11.3	0.667	34.7*
1000	*N. benthamiana*	44.0**	11.7**	0**	0**
	dip. *A. thaliana*	2.13**	6.50	NA	NA
	tet. *A. thaliana*	33.8*^††^	11.7*	0	30.0*

*n* = 4 in tetraploid *A. thaliana* Col*, n* = 3 in diploid *A. thaliana* Col and *N. benthamiana*, for each treatment

*NA* no flower formation was observed

*, **Significantly different from the control at 5% (*p* < 0.05) or 1% (*p* < 0.01) using Student *t* test

^†^^,††^Significantly different from diploid *A. thaliana* at 5% (*p* < 0.05) or 1% (*p* < 0.01) using Student *t* test

^a^Diploid, ^b^Tetraploid

The plant heights of diploid and tetraploid *A. thaliana* did not differ significantly in the control treatment (Table 3). However, tetraploid plants were significantly (*p* < 0.01) taller than the diploid ones in the 10 ml of 125–1000 mg/l TFMSA solution per plant treatments (Table 3). In the 10 ml of 500 and 1000 mg/l per plant treatments, flowers were observed in the tetraploid (Fig. S3), but not in the diploid (Fig. 3). The number of branches did not differ between the ploidy types at any TFMSA dosage, but flower formation appeared to be more tolerant to TFMSA in the tetraploid than in the diploid. For example, at 10 ml of 250 mg/l TFMSA per plant treatment, the number of siliques without seeds were more than four times higher in the tetraploid than in the diploid (Table 3). This suggests that the effect of TFMSA varied significantly in relation to ploidy; and furthermore, that higher-ploidy plants require more TFMSA to induce pollen sterility.

### Female functionality in cowpea after TFMSA treatment

TFMSA induced a high degree of pollen sterility in cowpea, but the female organs remained functional. The F_1_ seeds from crosses between a white-seeded accession IT97K-499-35 treated with TFMSA to induce male sterility and 29 untreated brown-seeded accessions germinated normally and produced normal F_2_ seeds, which were black (Table S2). The black seed coat indicated that the F_1_ plants were true hybrids, which is in agreement with Herniter et al. (2019). This suggests that the optimal TFMSA treatment regime induced pollen sterility in cowpea with no adverse effect on female functionality.

## Discussion

### Effect of TFMSA on dicotyledonous plants

In this study, we investigated the use of TFMSA as an effective male sterility inducer in cowpea and two model species, *A. thaliana* and *N. benthamiana*. The inability of treated plants to form self-pollinated pods, a detailed analysis of pollen viability via Alexander staining of a diverse set of genotypes and species, and evaluation of the effects of different TFMSA application rates confirmed the robustness of this technique. TFMSA induced male sterility in a dosage- and genotype-dependent manner. TFMSA solution applied at 30 ml with concentration of 1000 mg/l (w/v) per plant induced nearly 100% male sterility in cowpea, while a relatively lower concentration was effective in the model species (10 ml of 125–250 mg/l per plant for diploid *A. thaliana*, and 10 ml of 250–1000 mg/l per plant for tetraploid *A. thaliana* and for *N. benthamiana*).

The effectiveness of the TFMSA technique depends upon determining the most appropriate dose and timing of application for specific plant species. Unlike in the monocots sorghum and maize, for which the TFMSA solution was applied only to “youngest fully expanded leaves” (Hodnett and Rooney 2018), in this study the entire plant body was sprayed to induce pollen sterility. Loussaert (2004) reported that application of 200–500 µg of TFMSA per maize plant to the most recent fully expanded leaf effectively induced male sterility irrespective of the time of application. Similarly, Hodnett and Rooney (2018) and Kyanam et al. (2021) found that 30 mg TFMSA applied 34 days before flag leaf emergence, or 25 mg TFMSA in a split application (15 mg 55 days after planting, followed by 10 mg 9 days later) effectively induced male sterility in sorghum. In this study, the actual amount of the estimated TFMSA stayed on to cowpea at one-time application in IT86D-1010 was 10.4 mg per plant and 11.0 mg in IT97K-499-35. In this study, a one-time application of 30 ml of 1000 mg/l (w/v) TFMSA solution per plant applied 7 days before anthesis effectively induced total male sterility in 38 cowpea genotypes under field conditions. However, three of the tested accessions retained functional pollen after the single TFMSA treatment. This indicates that TFMSA might have genetic effect, which is in line with observations in sorghum and maize (Hodnett and Rooney 2018; Loussaert 2004). Three- or two-time applications at weekly intervals were more effective to induce male sterility. For a diverse collection of cowpea, therefore, the findings of this study suggest the optimum treatment is two applications of 30 ml of 1000 mg/l (w/v) TFMSA solution per plant and application (60 ml in total), applied a week apart, for complete male sterility induction. TFMSA induced 99% of pollen sterility which is enough for avoiding self-fertilization since our result suggests that 1% of the pollen grain cannot successfully form a pod.

Application of 10 ml of TFMSA at 125–250 mg/l for diploid *A. thaliana* and 250–1000 mg/l for *N. benthamiana* effectively induced complete male sterility under growth chamber conditions. In *A. thaliana*, the plant height and the number of the formed siliques were larger in the tetraploid than in the diploid type, likely as a result of a weakened effect of TFMSA in higher-ploidy plants. Ploidy level is correlated with cell size and pollen grain size (Altmann et al. 1994; Melaragno et al. 1993). Increased size of the cells may have reduced the effect of TFMSA for induction of male sterility in tetraploid *A. thaliana*.

### Durability of the effects of TFMSA

The induction of complete male sterility by treatment with TFMSA appears to be temporary, with reversion to pollen fertility as demonstrated by self-pollination. This reflects the duration of its activity, and is likely dependent on the dosage and timing of application (Boerman et al. 2019; Hodnett and Rooney 2018). Also, cowpea has indeterminate growth habit with the vegetative and reproductive stages occurring at the same time. This is a key factor why pollen functionality of cowpea can be restored in several weeks. In the current study, recovery of pollen fertility after TFMSA treatment was observed, and was directly related to the applied dosage. This observation supports the findings of Boerman et al. (2019) and Hodnett and Rooney (2018) regarding reversion of TFMSA induced male sterility, whereby cowpea can recover its pollen functionality after 2 months, especially from relatively lower doses. Proline is required for male fertility in *A. thaliana* (Mattioli et al. 2012). TFMSA induces male sterility in maize by limiting the amount of proline in the developing anthers (Loussaert 2004), and cowpea pollen function might also be attributable to the amount of proline transported in the developing anthers. In future studies, investigation of temporal proline accumulation in the pollen of TFMSA-treated cowpea and the model species may provide further insights. The observation of pod sets 7 days after the second treatment in cowpea suggested that some flowers had maintained fertility after the first TFMSA treatment. This result suggests that TFMSA was treated to small buds emerged after the first treatment had already passed the developmental stage where TFMSA can induce sterility. There is a timing threshold when TFMSA can induce sterility in the process of pollen development. This threshold is probably before proline transport and accumulation necessary to produce functional pollens. TFMSA treatment at an earlier stage, shortly before the reproductive stage starts, may prevent the emergence of fertile pollens. The lack of new pods during days 14–21 after the second TFMSA treatment suggested that TFMSA induced pollen sterility at a certain stage of pollen development in individual cowpea flowers.

### Impact of TFMSA on plant growth

The major drawbacks of using CHAs for effective male sterility induction are phytotoxicity and lack of specificity to induce male sterility without adverse effects on female functionality (Ghebrehiwot et al. 2015; Razzaq et al. 2016). Most of the CHAs belong to plant hormone groups, including auxins and auxin inhibitors, gibberellins and ethylene; they are toxic to the female structures or F_1_ seeds, and have a narrow window of timing for application (Colombo and Galmarini 2017). TFMSA is a common chemical and not yet found to be a plant hormone (Loussaert 2004; Moskalik and Astakhova 2022; Shainyan and Tolstikova 2013). TFMSA is classified as GHS system with code H315 + H320 (https://pubchem.ncbi.nlm.nih.gov/ghs/#_prec). No evidence of toxicity in animals or the environment is reported in the GHS classified as code H315 + H320 classification (Globally Harmonized System of Classification and Labelling of Chemicals (United Nations GHS Rev. 9, 2021)). The current study identified the dosage-dependent manner of TFMSA effects on plant growth and male functionality in cowpea, *A. thaliana* and *N. benthamiana*. The application of TFMSA, especially at relatively higher doses, significantly reduced growth of cowpea, *A. thaliana* and *N. benthamiana*. The effect was more pronounced in diploid *A. thaliana* than in tetraploid *A. thaliana* and *N. benthamiana*, which supports the hypothesis of species-specific and ploidy-dependent responses of plant growth and development to TFMSA.

TFMSA is known to suppress proline transport in plants, and proline has essential functions during the reproductive stage of plant growth (Biancucci et al. 2015; Loussaert 2004). Here, TFMSA was applied at the onset of the reproductive stage in each species. Cowpea is an indeterminate species with the vegetative and reproductive stages occurring at the same time, whereas *A. thaliana* and *N. benthamiana* are determinate plants. In cowpea, the reproductive stage starts after the vegetative stage when the flower buds start to emerge. The suppressive effect of TFMSA on functional pollen production in cowpea persisted for up to 2 months, or longer at high dosages, under greenhouse conditions. This suggests that TFMSA can induce nearly 100% pollen sterility, but the functionality of pollen grains may be restored and become viable several weeks after treatment due to the recovery of the plant from the effect of TFMSA. Under heat or dry conditions, with biotic and abiotic stresses, plants can accumulate proline (Hare and Cress 1997; Verbruggen and Hermans 2008). Thus, the effect of TFMSA might be weakened under those conditions. Kyanam et al. (2021) observed that TFMSA affects phenotypic traits in sorghum, including plant height reduction and flowering delay, but these effects were not severe enough to discourage its use as a breeding tool. For dicotyledonous species (cowpea, *A. thaliana* and *N. benthamiana*), appropriate TFMSA dosages that maintain male sterility without severe effects on plant growth and development may be useful for crossing purposes.

### Hybridization and breeding system with TFMSA

The two-time treatment with 30 ml of 1000 mg/l (w/v) TFMSA solution (60 ml per plant in total) had no adverse effect on cowpea female functionality, confirming its male-specific gametocidal effect (Loussaert 2004). This is in agreement with the effects of TFMSA in sorghum and maize (Boerman et al. 2019; Hodnett and Rooney 2018; Kyanam et al. 2021; Loussaert 2004). The hybrid cowpea plants did not show any growth impairment, suggesting that TFMSA did not have any genetic effect on female cowpea gametes. However, female functionality of TFMSA-treated plants was tested only with IT97K-499-35 in this study. Further investigation of female functionality is needed with other genotypes in future studies. TFMSA may be useful for future large-scale crossing experiments in a wide range of species to shuffle genes in large population groups to create diverse hybrid populations.

## Conclusions

TFMSA applied at the optimum dosage and timing induced pollen sterility in a genetically diverse group of cowpea accessions, with no adverse effect on female functionality. The specific induction of pollen sterility by TFMSA, without compromising female functionality, is highly promising for creating large numbers of hybrids in self-pollinated species. To the best of our knowledge, this is the first report of induction of male sterility in cowpea and the two model species (*A. thaliana* and *N. benthamiana*) by the use of TFMSA as a male-specific gametocide. Since there are few male sterility genes in cowpea, the temporary male sterility induced by the TFMSA technique in this study may be a useful tool for pollination control. This new breeding technique may also have significant implications for breeding and hybrid seed production in other self-pollinated species.

### Author contribution statement

TI designed the experiments. YS performed the experiments. YS, TI and BU wrote the paper.

## Supplementary Information

Below is the link to the electronic supplementary material.

Supplementary file1 (DOCX 5258 kb)

## Data Availability

All data generated in this study on which the conclusions are based are included in this published article.
