# Supplementary material for: Chemical emasculation in cowpea (Vigna unguiculata (L.) Walp.) and dicotyledonous model species using trifluoromethanesulfonamide (TFMSA)
Source: Plant Reprod. 2023 May 25;36(3):273–84. doi: 10.1007/s00497-023-00469-4 (PMC10363044; doi:10.1007/s00497-023-00469-4)
Supplement: Supplementary file 1 — Supplementary file1 (DOCX 5258 kb) [file 497_2023_469_MOESM1_ESM.docx]

**Fig. S1**

Multiple applications of 30 ml of 1000 mg/l TFMSA per plant to cowpea. (a) TFMSA was applied from one to five times at intervals of one week until anthesis of the control. (b) Treated IT97K-499-35 cowpea plants photographed when the control plants started flowering. Blue arrows indicate the TFMSA treatment.

**Fig. S2**

Anthers from 42 cowpea accessions after treatment with 30 ml of 1000 mg/l TFMSA per plant in the field. The photomicrographs are grouped by degree of sterility. Complete: full of sterile pollen, Partial: half of sterile pollen, Non: no sterile pollen. Scale bars, 500 μm

**Fig. S3**

Effect of different TFMSA dosage on tetraploid *A. thaliana* at 32 days after the second treatment


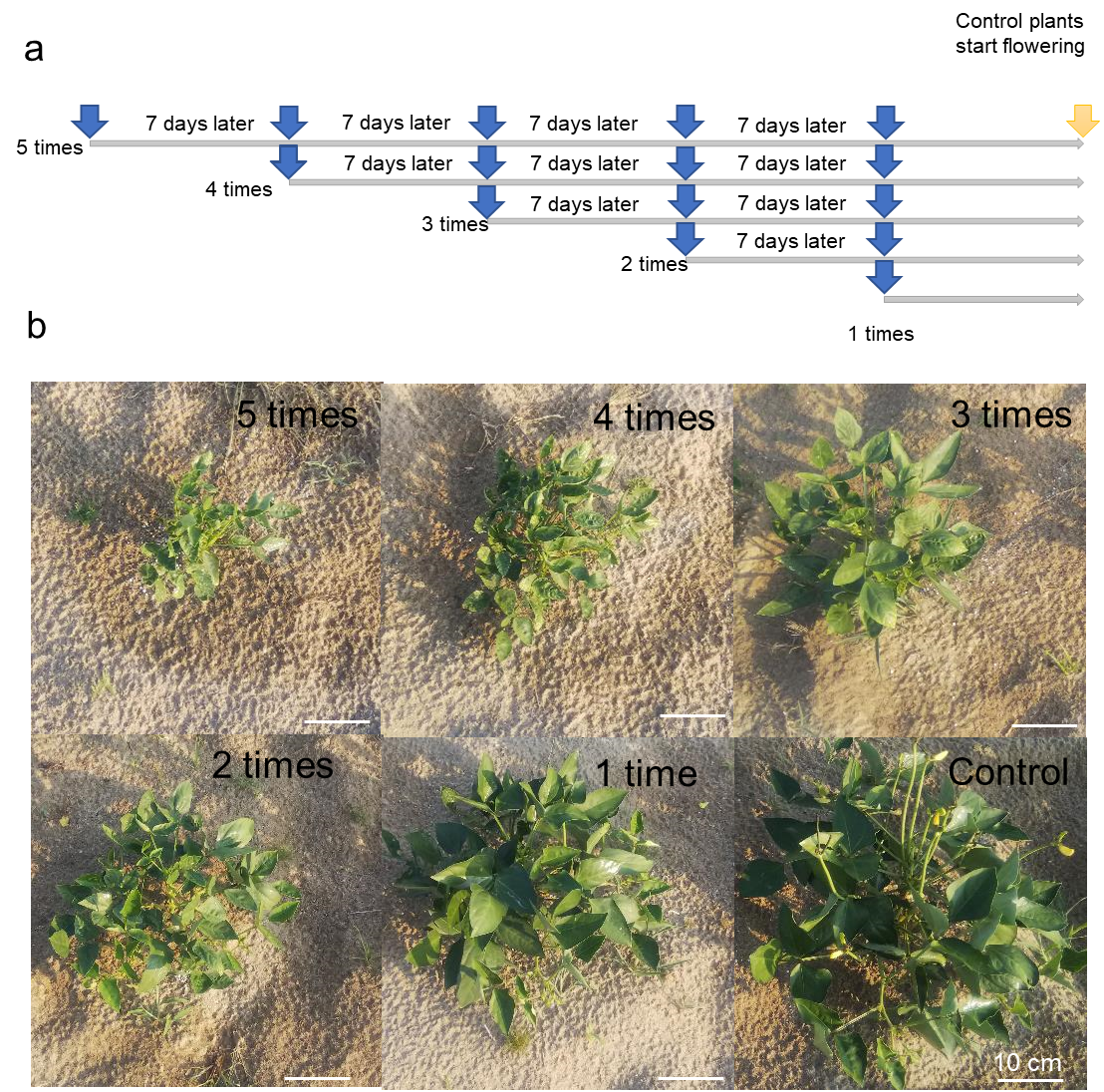


**Fig. S1**


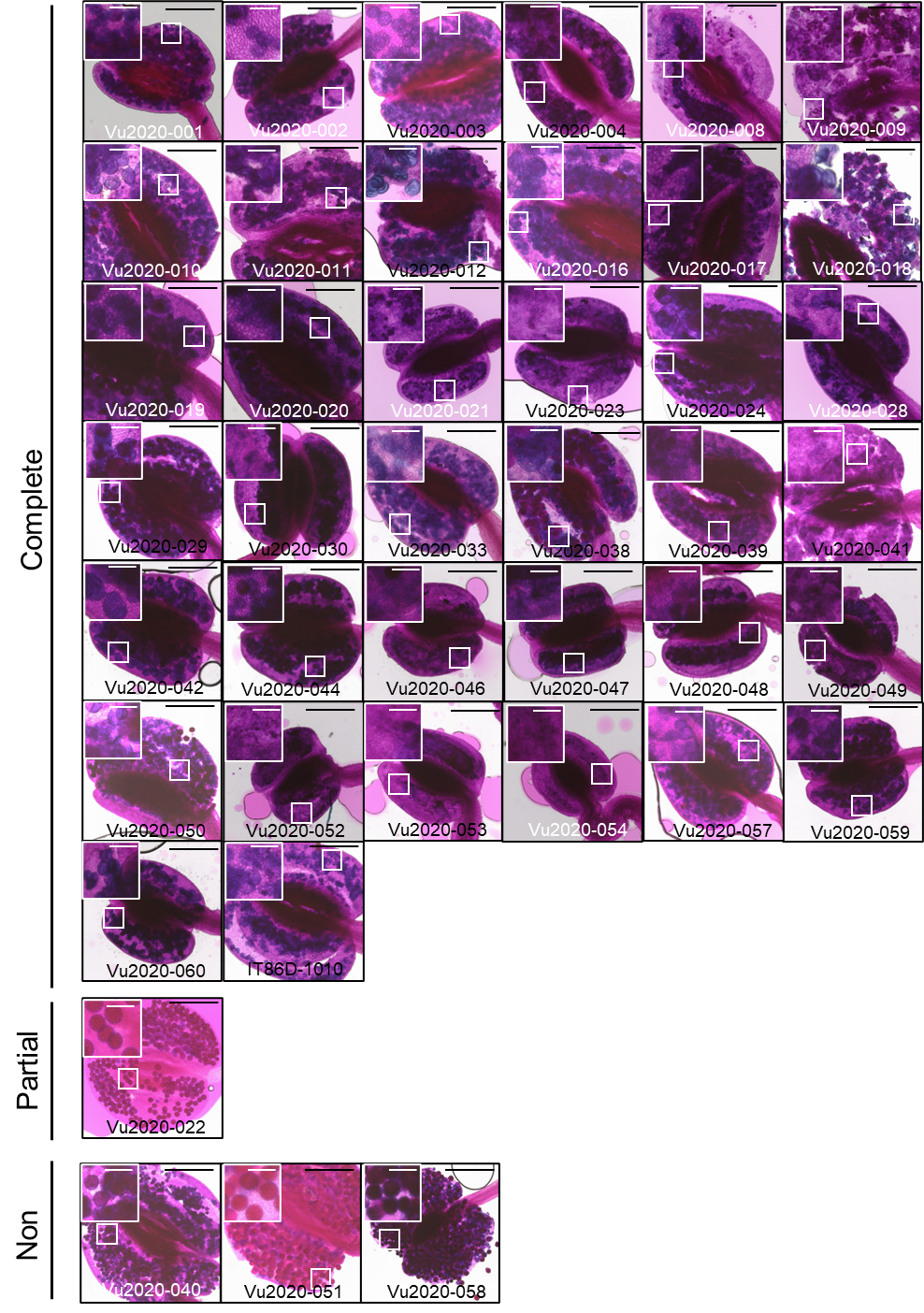


**Fig. S2**

**
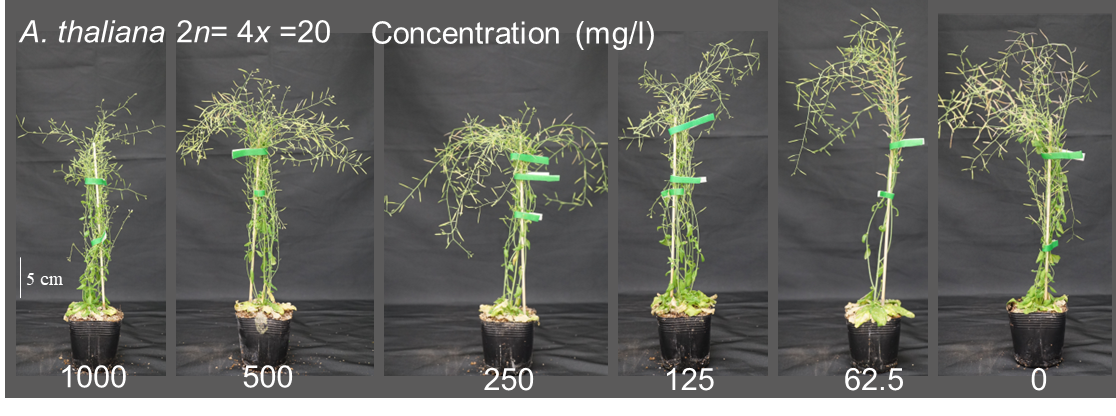
**

**Fig.S3**

**Table S1** Plant materials used in this study

| Plant material | Accession ID |
| --- | --- |
| *Arabidopsis thaliana* Columbia |  |
| Diploid | 140931 |
| Tetraploid | N3151 |
| *Nicotiana benthamiana* | N/A^a^ |
| *Vigna unguiculata* ssp. *unguiculata* |  |
| IT86D-1010 | IT86D-1010 |
| IT97K-499-35 | IT97K-499-35 |
| Vu2020-001 | 53841 |
| Vu2020-002 | 74826 |
| Vu2020-003 | 74828 |
| Vu2020-004 | 80035 |
| Vu2020-005 | 76525 |
| Vu2020-006 | 31615 |
| Vu2020-007 | 110719 |
| Vu2020-008 | 31624 |
| Vu2020-009 | 87823 |
| Vu2020-010 | 78862 |
| Vu2020-011 | 85514 |
| Vu2020-012 | 31622 |
| Vu2020-013 | 73010 |
| Vu2020-014 | 42923 |
| Vu2020-015 | 42927 |
| Vu2020-016 | 42929 |
| Vu2020-017 | 42931 |
| Vu2020-018 | 85409 |
| Vu2020-019 | 85410 |
| Vu2020-020 | 85416 |
| Vu2020-021 | 85424 |
| Vu2020-022 | 85432 |
| Vu2020-023 | 85455 |
| Vu2020-024 | 85463 |
| Vu2020-025 | 85467 |
| Vu2020-026 | 85471 |
| Vu2020-027 | 85472 |
| Vu2020-028 | 85475 |
| Vu2020-029 | 74832 |
| Vu2020-030 | 74833 |
| Vu2020-031 | 74837 |
| Vu2020-032 | 74843 |
| Vu2020-033 | 104293 |
| Vu2020-034 | 104295 |
| Vu2020-035 | 104297 |
| Vu2020-036 | 104306 |
| Vu2020-037 | 222494 |
| Vu2020-038 | 81552 |
| Vu2020-039 | 81592 |
| Vu2020-040 | 81595 |
| Vu2020-041 | 31654 |
| Vu2020-042 | 31659 |
| Vu2020-043 | 31661 |
| Vu2020-044 | 31662 |
| Vu2020-045 | 31671 |
| Vu2020-046 | 31674 |
| Vu2020-047 | 80029 |
| Vu2020-048 | 236697 |
| Vu2020-049 | 236702 |
| Vu2020-050 | 236745 |
| Vu2020-051 | 81556 |
| Vu2020-052 | 233358 |
| Vu2020-053 | 233361 |
| Vu2020-054 | 233372 |
| Vu2020-055 | 89230 |
| Vu2020-056 | 251276 |
| Vu2020-057 | 257502 |
| Vu2020-058 | 257553 |
| Vu2020-059 | 257554 |
| Vu2020-060 | N/A |

^a^ Not available.

**Table S2** Seeds generated from crosses of TFMSA-treated female parent (IT97K-499-35, white seed coat) × untreated accessions.

| Male parent  (brown seed coat) | Number of seeds per F1 pod  (one pod from each accession) |
| --- | --- |
| Vu2020-2 | 10 |
| Vu2020-4^a^ | 1 |
| Vu2020-8 | 2 |
| Vu2020-10 | 11 |
| Vu2020-11 | 5 |
| Vu2020-12 | 2 |
| Vu2020-14 | 1 |
| Vu2020-17^a^ | 7 |
| Vu2020-18 | 2 |
| Vu2020-21 | 2 |
| Vu2020-23 | 11 |
| Vu2020-24^a^ | 5 |
| Vu2020-26 | 6 |
| Vu2020-27 | 6 |
| Vu2020-28 | 8 |
| Vu2020-31 | 11 |
| Vu2020-32 | 11 |
| Vu2020-33 | 2 |
| Vu2020-36 | 5 |
| Vu2020-39 | 7 |
| Vu2020-40 | 10 |
| Vu2020-42 | 9 |
| Vu2020-48^a^ | 8 |
| Vu2020-49 | 3 |
| Vu2020-53 | 1 |
| Vu2020-54 | 8 |
| Vu2020-55 | 11 |
| Vu2020-59^a^ | 8 |
| Vu2020-60 | 10 |

^a^ No F2 seed was available from F1 plants; all other F2 seeds were black.

**Table S3** The exact amount of the TFMSA solution sprayed onto plants

| Species | Applied solution (ml) | Mean Fresh weight before treatment (g) | Mean increased weight after treatment (g) | Estimated TFMSA amount / plant fresh weight (mg/g) |
| --- | --- | --- | --- | --- |
| IT86D-1010 | 30 | 83.0 ± 7.27 | 11.0 ± 1.81 | 0.13 |
| IT97K-499-35 | 30 | 81.8 ± 18.8 | 10.4 ± 1.12 | 0.13 |
| *N. benthamiana* | 10 | 9.73 ± 1.11 | 2.72 ± 0.239 | 0.280 |

n=5 for IT86D-1010 and *N. benthamiana*, n=4 for IT97K-499-35 Estimated TFMSA amount is calculated when the concentration is 1000mg/l.

**Table S4** Number of formed pods counted on 0, 7, 14, 21, and 61 days after second treatment of TFMSA with different dosage.

|  | Mean pod number on the days after second treatment | | | | |
| --- | --- | --- | --- | --- | --- |
| Concentration (mg/l) | 0 | 7 | 14 | 21 | 61 |
| 0 | 4.0 ± 3.3 | 5.3 ± 2.1 | 4.3 ± 2.1 | 4.7 ± 2.5 | 9.7 ± 3.8 |
| 62.5 | 2.7 ± 1.7 | 2.7 ± 0.9 | 2.3 ± 1.2 | 2.3 ± 1.2 | 9.3 ±4.0* |
| 125 | 3.3 ± 0.5 | 4.0 ± 0.8 | 3.0 ± 0.8 | 3.7 ± 0.9 | 8.7 ± 5.0 |
| 250 | 3.0 ± 1.6 | 4.0 ± 0.8 | 4.0 ± 0.8 | 3.7 ± 0.9 | 9.0 ± 2.8* |
| 500 | 2.0 ± 1.4 | 3.3 ± 2.5 | 2.7 ± 2.1 | 3.0 ± 2.2 | 5.3 ± 5.6 |
| 1000 | 2.7 ± 0.9 | 4.0 ± 2.9 | 3.3 ± 2.6 | 3.3 ± 2.6 | 2.7 ± 3.1 |

n = 2 or 3, for each dosage. * Significantly different from the Day 0 at 10% (P<0.1) using Student t-test. ± indicates standard deviation
